# Sequence Variation Analysis of Epstein-Barr Virus Nuclear Antigen 1 Gene in the Virus Associated Lymphomas of Northern China

**DOI:** 10.1371/journal.pone.0140529

**Published:** 2015-10-13

**Authors:** Lingling Sun, Zhenzhen Zhao, Song Liu, Xia Liu, Zhifu Sun, Bing Luo

**Affiliations:** 1 Department of Pathology, Affiliated Hospital of Qingdao University, Qingdao, China; 2 Department of Medical Microbiology, Qingdao University Medical College, Qingdao, China; 3 Department of Health Sciences Research, Mayo Clinic, Rochester, Minnesota, United States of America; The University of North Carolina at Chapel Hill, UNITED STATES

## Abstract

Epstein-Barr virus (EBV) nuclear antigen 1 (EBNA1) is the only viral protein expressed in all EBV-positive tumors as it is essential for the maintenance, replication and transcription of the virus genome. According to the polymorphism of residue 487 in EBNA1 gene, EBV isolates can be classified into five subtypes: P-ala, P-thr, V-val, V-leu and V-pro. Whether these EBNA1 subtypes contribute to different tissue tropism of EBV and are consequently associated with certain malignancies remain to be determined. To elucidate the relationship, one hundred and ten EBV-positive lymphoma tissues of different types from Northern China, a non-NPC endemic area, were tested for the five subtypes by nested-PCR and DNA sequencing. In addition, EBV type 1 and type 2 classification was typed by using standard PCR assays across type-specific regions of the EBNA3C genes. Four EBNA1 subtypes were identified: V-val (68.2%, 75/110), P-thrV (15.5%, 17/110), V-leuV (3.6%, 4/110) and P-ala (10.9%, 12/110). The distribution of the EBNA1 subtypes in the four lymphoma groups was not significantly different (*p* = 0.075), neither was that of the EBV type 1/type 2 (*p* = 0.089). Compared with the previous data of gastric carcinoma (GC), nasopharyngeal carcinoma (NPC) and throat washing (TW) from healthy donors, the distribution of EBNA1 subtypes in lymphoma differed significantly (*p* = 0.016), with a little higher frequency of P-ala subtype. The EBV type distribution between lymphoma and the other three groups was significantly different (*p* = 0.000, *p* = 0.000, *p* = 0.001, respectively). The proportion of type 1 and type 2 mixed infections was higher in lymphoma than that in GC, NPC and TW. In lymphomas, the distribution of EBNA1 subtypes in the three EBV types was not significantly different (*p* = 0.546). These data suggested that the variation patterns of EBNA1 gene may be geographic-associated rather than tumor-specific and the role of EBNA1 gene variations in tumorigenesis needs more extensive and deep explorations.

## Introduction

Epstein–Barr virus (EBV) is an oncogenic virus that infects >90% of the global population. Latent EBV infection is associated with a variety of lymphoid and epithelial malignancies including Burkitt’s lymphoma, classical Hodgkin lymphoma (cHL), diffuse large B cell lymphoma (DLBCL), natural killer (NK)/T-cell lymphoma, nasopharyngeal carcinoma (NPC) and gastric carcinoma (GC)[1, 2]. However, the exact role of EBV in tumorigenesis remains unclear. Despite the ubiquity of EBV infection, only a small proportion of individuals develop EBV-associated neoplasms and the incidence of these tumors varies in different geographic regions. This variability may be contributed by differences in human host genetic, environmental, or viral factors. The possibility of particular substrains of EBV responsible for different tissue tropisms and development of certain EBV-associated malignancies has been long suspected. Substantial genetic sequence variations in EBV have been found among EBV isolates on the EBV genome, but the role of these variations has yet to be elucidated [3].

During EBV latent infection, EBV persists in host cells and expresses a limited set of viral gene products, including EBV nuclear antigens (EBNAs), latent membrane proteins (LMPs) and EBV-encoded small noncoding RNAs (EBERs). EBNAs include EBNA1, EBNA2, EBNA3(3A, 3B, 3C) and EBNA LP. EBNA1 is a 641 amino acid protein, consistently expressed in all EBV-associated malignant tissues [4, 5]. It is essential for the maintenance, replication and transcription of the EBV genome in host cells. In addition, EBNA1 may affect cellular proteins and signal pathways involved in cell survival and proliferation so that it plays a critical role in the development and/or progression of EBV-associated tumors [6]. The EBNA1 protein is composed of unique amino-terminal (residues 1–89) and carboxyl-terminal (residues 327–641) domains linked by a large Gly-Ala repeat (residues 90–326) [7]. Most reported substitutions were identified in the carboxyl-terminal, which contains the dimerization domain, DNA binding domain, and transactivation domain [8]. Sequence variations may have a larger impact on the function of these important domains, and consequently affect DNA replication, transcription or oncogenic potential of the virus. According to the amino acid at residue 487 in EBNA1 gene, EBV isolates can be divided into five subtypes including two prototypes (P-ala and the closely related P-thr) and three variants (V-val, V-leu and V-pro), in which the AA 487 site was alanine, threonine, valine, leucine and proline, respectively [9, 10].

The sequence variation of EBNA1 gene has been widely explored, but whether EBNA1 subtypes were tumor-specific or only geographically restricted remains elusive. Some believe that EBNA1 variations may contribute to different tissue tropisms of EBV and are associated with certain malignancy development [9–12], while others argue that they only reflect region-restricted polymorphisms [13–16]. The controversial results may be caused by a small sample size, limited geographic region and disease status, and control sample selection [17].

Most studies on EBNA1 variations in China have been limited to NPC in Southern China, the NPC endemic area, and the results suggest that the V-val is the only EBNA1 subtype in NPC and is preferentially associated with NPC [10, 12]. To characterize the variations of EBNA1 in non-NPC endemic area in China, we previously analyzed the polymorphisms of EBNA1 gene in EBV-associated gastric carcinoma (EBVaGC), NPC and throat washing samples from healthy donors in Northern China, and concluded that there was no evidence that particular EBNA1 subtypes were preferentially associated with either EBVaGC or NPC [18]. However, EBNA1 variation in lymphoma, another malignancy closely related to EBV, has not yet been extensively studied in China. In order to explore the association between EBNA1 subtypes and EBV-associated lymphoma, we investigated sequence variations of EBNA1 gene in 110 EBV-positive lymphoma biopsies from Northern China and compared the results with those from our previous and other reports. The EBNA3C variants (EBV type1/type2) were also analyzed to demonstrate the association of the two different classifications.

## Materials and Methods

### Specimens and DNA extraction

This study followed guidelines under Declaration of Helsinki and was approved by the Medical Ethics Committee of Qingdao University Medical College. Written informed consents were obtained from all the study participants.

Six hundreds and twenty-two paraffin-embedded lymphoma tissues were collected from the Department of Pathology of Affiliated Hospital of Qingdao University in Shandong Province, Northern China, a non-NPC endemic area. EBV infection in lymphomas was determined by EBV-encoded small RNA (EBER) 1 in situ hybridization, as described previously [19]. One hundred and ten EBV-positive lymphomas, including 59 nasal natural killer/T cell lymphomas (NK/T), 35 Hodgkin’s lymphomas (HL), 5 diffuse large B cell lymphomas (DLBCL), and 11 T cell lymphomas, were obtained.

DNA from the paraffin-embedded lymphoma tissues was extracted using QIAamp DNA FFPE Tissue Kit (QIAGEN GmbH, Hilden, Germany).

### Amplification of EBNA1 C-terminal domain

Nested-polymerase chain reaction (nested-PCR) technique was performed to amplify the C-terminal region of EBNA1. Specific oligonucleotide primers were designed. The sequences and positions of these primers are EBNA1-1, 5’-TAGTCAGTCATCATCATCCG-3’ (B95-8 coordinate 109104–109123), EBNA1-2, 5’-GGGATTTATTCTTTAGTGCG-3’ (B95-8 coordinate 109946–109927); EBNA1-3, 5’-GCCATTTTTCCACCCTGTAG-3’ (B95-8 coordinate 109158–109177), EBNA1-4, 5’-ATTGAGGGCGTCTCCTAACA-3’ (B95-8 coordinate 109902–109883). EBNA1-1 and EBNA1-2 were used in the first round PCR, and EBNA1-3 and EBNA1-4 were used in the second round.

The first round of PCR was performed in a total volume of 25 μl, containing 1×PCR reaction buffer, 0.2 mM of each deoxyribonucleotide triphosphate, 1.5 mM of MgCl_2_, 0.4 μM of each primer, 1 U PfuTaq polymerase (TaKaRa Biotechnology Co., Ltd., Kyoto, Japan) and 100 ng of genomic DNA. PCR amplification was performed with an initial denaturation at 94°C for 5 min; 35 cycles of denaturation at 94°C for 30 s, annealing at 55°C for 30 s, extension at 72°C for 1 min; and a final elongation step at 72°C for 10min. The products of the first round PCR were amplified in the second round PCR with internal primers EBNA1-3 and EBNA1-4 in a total volume of 30 μl and the final concentration of each composition was the same with the first round of PCR.

In each set of PCR, DNA from EBV-positive B95-8 cell lines and EBV-negative Ramos cell lines were used as positive and negative controls, respectively. The PCR products were analyzed by electrophoresis through a 1.2% agarose gel. After electrophoresis, the gels were stained with ethidium bromide and observed under the UV light transilluminator.

### EBNA1 C-terminal domain sequencing

27 μl products of the second round of PCR were directly sequenced in both directions with primers EBNA1-3 and EBNA1-4 by means of a Prism ready reaction Dyedeoxy terminator cycle sequencing kit (Applied Biosystems, Foster, USA). In selected samples with multiple signals at the same nucleotide positions, the final EBNA1 amplimers were subcloned into TA-cloning vectors (pMD18-T vector, TaKaRa Biotechnology Co., Ltd., Kyoto, Japan), and independent clones were subjected to DNA sequencing with M13R and M13F primer for each specimen. The EBNA1 gene sequence data were checked for homology by using BLAST (National Center for Biotechnology Information) and were compared with the B95-8 strain (GenBank accession no.V01555). Alignments between sequences were analyzed using DNA Star software (DNASTAR, Inc., version 5.0).

### Determination of EBNA1 Subtypes

According to previous studies [9, 10], we used AA 487 as the signature residue combined with other common substitutions to classify the EBNA1 sequence variation patterns. The sequence of P-ala was identical to that of the B95-8 strain. V-val was just the same as that previously reported by Bhatia et al. [9]and Gutiérrez et al. [10]. P-thrV and V-leuV were designated because they were somewhat different from the common P-thr and V-leu respectively. Mixed EBV infection was determined when multiple signals were detected at the same nucleotide positions.

### Definition of EBV type 1/type 2

Definition of EBV type 1/type 2 was performed by using standard PCR assays across type-specific regions of the EBNA3C genes, as previously reported [20]. The EBV-positive cell lines B95-8 and P3HR-1 were used as controls for EBV type 1 and EBV type 2, respectively. The PCR products were separated on a 2% agarose gel and visualized by ethidium bromide staining.

### Statistical analysis

The distribution of EBNA1 subtypes and EBV type 1/type 2 among four different groups of lymphoma or between lymphoma and our previous detected samples (EBVaGC, NPC and TW from healthy donors) were compared, and the association of EBV type and EBNA1 polymorphisms was analyzed. The χ^2^ test and Fisher’s exact tests were performed to determine the distribution difference of the EBV variations. The results were considered to be statistically significant when p<0.05. Statistical analyses were conducted using SPSS 19.0 statistical software (SPSS, Chicago, IL).

## Results

### C-terminal sequence patterns of EBNA1

The gene fragment analyzed in the study was the C-terminal region of EBNA1 from amino acid (AA) 404 to 641 (nt 109159–109875) which covers most of the sequence variations reported in the literature. All the 110 EBV-positive lymphoma samples including 59 NK/T, 35 HL, 5 DLBCL and 11 T cell lymphomas were successfully amplified and sequenced for the EBNA1 gene. We used AA 487 as the signature residue to classify the sequence variation patterns. The sequences with identical consensus mutations were arranged into one group. Analysis showed the presence of a single EBNA1 sequence in 108 of 110 cases (97.3%), whereas the remaining 2 samples (2.7%) displayed dual EBNA1 sequences within the same tumor tissue (Table 1). Each case with double signals at several EBNA1 nucleotide positions in direct sequencing was further confirmed by T-A cloning and sequencing for multiple clones, and two different, independent EBNA1 sequences were found in every case. Four EBNA1 subtypes were detected, and subtype V-pro was not found.

**Table 1:** The distribution of EBNA1 variants and EBV types in NK/T, HL, DLBCL and T cell lymphoma.

	NK/T(n = 59)	HL(n = 35)	DLBCL(n = 5)	T(n = 11)
EBNA1 subtypes				
V-val	36(61.0%)	28(80.0%)	4(80%)	7(63.6%)
P-thrV	9(15.3%)	6(17.1%)	0	2(18.2%)
P-ala	10(16.9%)	1(2.9%)	0	1(9.1%)
V-leuV	4(6.8%)	0	0	0
V-val + P-ala	0	0	1(20%)	1(9.1%)
EBV types				
Type 1	49(83.1%)	23(65.7%)	4(80%)	5(45.5%)
Type 2	2(3.4%)	4(11.4%)	0	1(9.0%)
Type 1+2	8(13.5%)	8(22.9%)	1(20%)	5(45.5%)

NK/T: natural killer/T cell lymphoma. HL: Hodgkin's lymphoma. DLBCL: diffuse large B cell lymphoma; T: T cell lymphoma.

**Fig 1:**
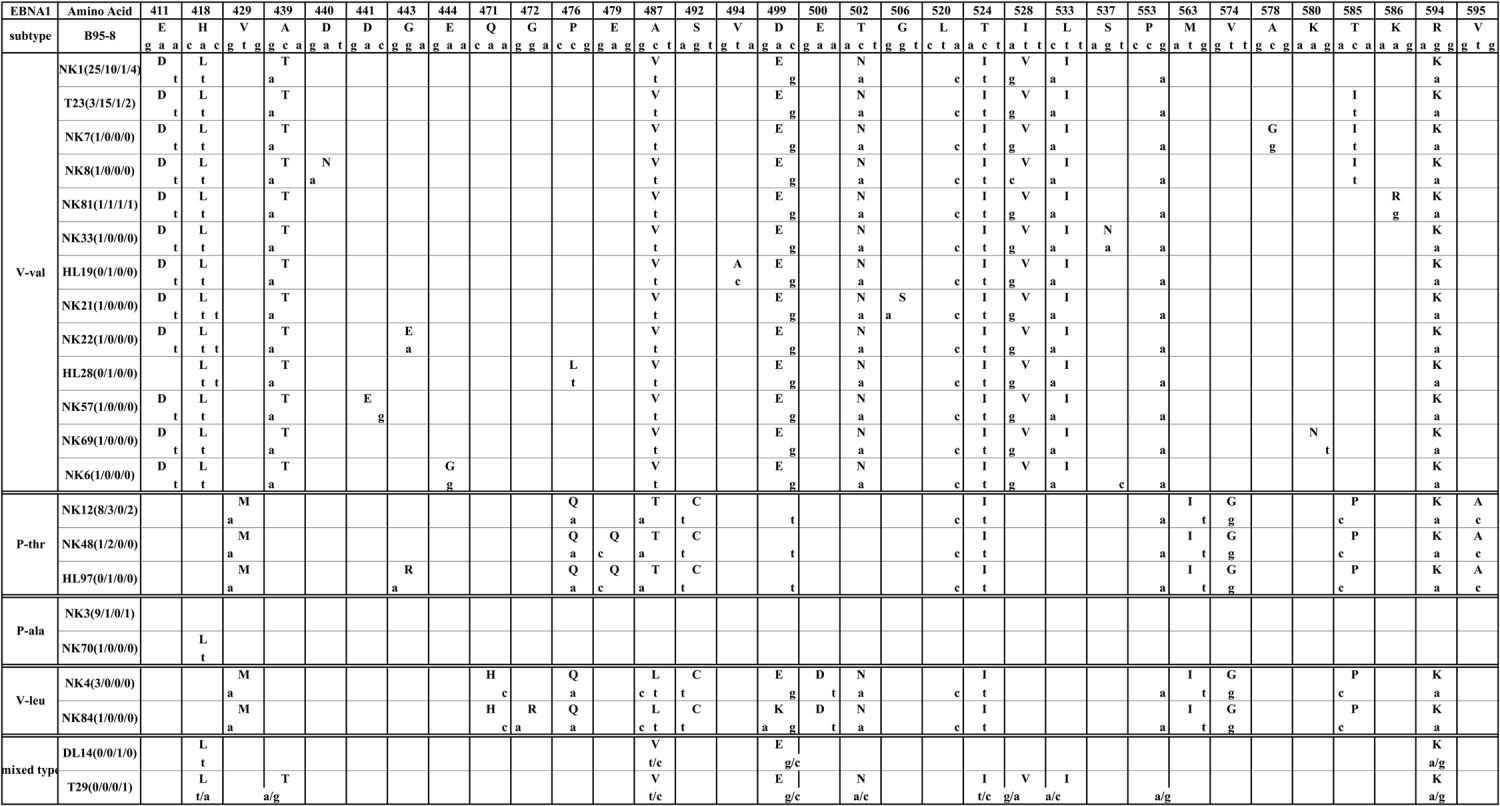
EBNA1 sequence variations in four groups of lymphoma. The numbers across the top correspond to the amino acid positions under which the B95-8 prototype amino acid and nucleotide sequence are listed. Different patterns are noted to the left column, while the specimens showing identical sequences to each other are listed by a representative isolate in the second column. The followed numbers in the parentheses denote the amount of the identical sequences from NK/T, HL, DLBCL, and T cell lymphoma, respectively. The small letters denote the nucleotide, and the amino acids are denoted by capital letters. NK: nasal natural killer/T cell lymphoma; HL: Hodgkin’s lymphoma; DL: diffuse large B cell lymphoma; T: T cell lymphoma. Some rare sporadic mutations are not shown in Fig 1.

V-val subtype was the most common pattern with a frequency of 68.2% (75/110). This pattern (represented by NK1 in Fig 1) contained 12 consensus sequence changes, including 10 AA changes and two silent changes. The 10 AA changes were at residues 411 (Glu→Asp), 418 (His→Leu), 439 (Ala→Thr), 487 (Ala→Val), 499 (Asp→Glu), 502 (Thr→Asn), 524 (Thr→Ile), 528 (Ile→Val), 533 (Leu→Ile) and 594 (Arg→Lys). The 2 silent variations were at residues 520 (CTA→CTC) and 553 (CCG→CCA). In this group, partial isolates showed one or more additional changes except for the consensus sequence changes. There were 23 isolates (representative by T23) with a remarkable substitution at residue 585 (Thr→Ile), appearing in 5 NK/T (8.5%, 5/59), 15 HL (42.9%, 15/35), 1 DLBCL (20%, 1/5) and 2 T cell lymphomas (18.2%, 2/11). This subvariant of V-val was also observed in 21 samples (1 GC, 12 NPC and 8 TW) in our previous study. Another less frequent simultaneous substitution happened at residue 586 (Lys→Arg) in 1 NK/T, 1 HL, 1 DLBCL, 1 T cell lymphoma and also in 1 previous TW sample.

The second common pattern (represented by NK12 in Fig 1) was found in 15.5% (17/110) of specimens. It has 13 consensus sequence changes, including 10 coding changes at residues 429 (Val→Met), 476 (Pro→Gln), 487 (Ala→Thr), 492 (Ser→Cys), 524 (Thr→Ile), 563 (Met→Ile), 574 (Val→Gly), 585 (Thr→Pro), 594 (Arg→Lys) and 595 (Val→Ala) and 3 silent changes at residues 499 (GAC→GAT), 520 (CTA→CTC) and 553 (CCG→CCA). The consensus sequence of this pattern was different from that of the P-thr subtype designated by Bhatia et al. [9], with additional changes at residues 476, 492, 499 and 520 and absence of the change at residue 525. We termed this pattern P-thr variant (P-thrV). 4 isolates (representative by NK48 in Fig 1) in the P-thrV group carried one additional substitution at residue 479 (Glu→Gln), in which 1 sample (HL97) carried one more substitution at residue 443 (Gly→Arg). The substitution at residue 479 (Glu→Gln) was present in 1 NK/T, 3HL and previously found in 1 EBVaGC, 3NPC and 2 TW from healthy donors.

P-ala subtype (represented by NK3 in Fig 1) was the third pattern and detected in 10.9% (12/110) of the specimens. The sequence of this subtype was the same as that of B95-8 except that one specimen (NK70) had one substitution at residue 418 (His→Leu). Interestingly, we didn’t identify EBNA1 gene sequence identical to the prototype B95-8 in NPC, EBVaGC and TW previously, instead obtained a subvariant with Ala at residue 487 and five AA substitutions at residues 439 (Ala→Thr), 499 (Asp→Glu), 24 (Thr→Ile), 588 (Ala→Pro) and 594 (Arg→Lys), as well as a silent base change at codon 553 (CCG→CCA), which we named P-ala variant (P-alaV).

V-leuV subtype (represented by NK4 in Fig 1) was detected in 3.6% (4/110) of the specimens. It had 15 mutations relative to B95-8. AA 471 (Gln→His), 487 (Ala→Leu) and 500 (Glu→Asp) exclusively existed in V-leu. AA 429 (Val→Met), 476 (Pro→Gln), 492 (Ser→Cys), 563 (Met→Ile), 574 (Val→Gly) and 585 (Thr→Pro) were identical to P-thrV. AA 499 (Asp→Glu) and 502 (Thr→Asn) co-existed in V-leu and V-val. Two AA substitutions including AA 524 (Thr→Ile) and AA594 (Arg→Lys) and two silent mutations including codons 520 (CTA→CTC) and 553 (CCG→CCA) were identified in V-leu, P-thrV and V-val. The consensus sequence between AA 466 and 527 was almost the same as the V-leu variant described by Bhatia et al. (1996) except for the absence of substitution at codon 525 (Ala→Gly) and the presence of AA His (CAC) instead of Glu (GAA) at residue 471; in view of these differences, it was classified as V-leu variant (V-leuV).

Two samples contained two distinct sequences in each sample simultaneously. One DLBCL (DL14) and one T cell lymphoma (T29) were both coinfected with V-val and P-ala (Table 1, Fig 1) respectively.

### Distribution of EBNA1 subtypes in different samples

Table 1 shows the distribution of EBNA1 subtypes in four subtypes of lymphoma (NK/T, HL, DLBCL, T cell lymphoma). In each group, the most common subtype was V-val without exception. Of the 108 cases with single EBNA1 sequence, 75 (36 NK/T, 28 HL, 4 DLBCL and 7 T cell lymphoma samples) carried V-val subtype, 17 (9 NK/T, 6 HL, 2 T cell lymphoma) carried P-thrV subtype, 12 (10 NK/T, 1 HL, 1 T cell lymphoma) carried P-ala subtype and 4 (4 NK/T) carried V-leuV subtype. The distribution of EBNA1 subtypes among HL, NK/T, DLBCL and T cell lymphoma was not significantly different (*p* = 0.075).

In order to get more comprehensive information to elucidate the relationship between EBNA1 variation and disease, we compared these results with that from NPC, EBVaGC and TWs from healthy donors which we previously generated in our laboratory [18]. The results were summarized in Table 2. V-val was the most common subtype in each group followed by P-thrV. The distribution of EBNA1 subtypes had significant difference between lymphoma, NPC, EBVaGC and TWs (*p* = 0.016). The P-ala/P-alaV subtype appeared more frequently in lymphoma samples than other three groups.

**Table 2:** The distribution of EBNA1variants and EBV types in lymphomas, nasopharyngeal carcinomas, EBVaGCs and throat washing samples from healthy donors.

	Lymphoma(n = 110)	NPC(n = 41)	EBVaGC(n = 41)	TWs(n = 55)
EBNA1 variants				
V-val	75(68.2%)	30 (73.2%)	32 (78.0%)	34 (61.8%)
P-thrV	17(15.5%)	10 (24.4%)	5 (12.2%)	15 (27.3%)
V-leuV	4(3.6%)	1 (2.4%)	2 (4.9%)	1 (1.8%)
P-ala	12(10.9%)	0	0	1 (1.8%)
V-val + P-ala	2(1.8%)	0	0	1 (1.8%)
V-val + P-thrV	0	0	1 (2.4%)	3 (5.5%)
V-val + V-leuV	0	0	1 (2.4%)	0
EBV types				
Type 1	81(73.6%)	34(82.9%)	36(87.8%)	41(74.5%)
Type 2	7(6.4%)	7(17.1%)	5(12.2%)	12(21.8%)
Type1+2	22(20%)	0	0	2(3.7%)

NPCs: nasopharyngeal carcinomas; EBVaGCs: EBV-associated gastric carcinomas; TWs: throat washings from healthy donors.

### Typing of EBV strains present in samples

All the 110 EBV-positive lymphoma biopsies were successfully amplified for EBV type 1/type 2 designations. Type 1 EBV alone was present in 81 (73.6%) lymphoma samples and type 2 EBV alone was found in 7 (6.4%) samples. Twenty two (20%) displayed dual type 1 and type 2 EBV strains infection. The frequency of the EBV types in HL, NK/T, DLBCL and T cell lymphoma was showed in Table 1. There was no significant difference for the distribution of type1, 2 and type 1+2 in four groups of lymphomas (*p* = 0.089).

Furthermore, we compared the distribution of EBV types among lymphoma, NPC, EBVaGC and TWs. The result was summarized in Table 2. Type 1 and 2 coinfected samples accounted for 20% (22/110) in lymphomas, but 3.7% (2/55) in TWs, none in NPC and EBVaGC. There was significantly difference about the distribution of EBV types in four group of cases (*p* = 0.000) and further comparison of the lymphoma group with NPC, EBVaGC and TWs demonstrated that it was statistically significant difference in all the comparisons (*p* = 0.000, *p* = 0.000, *p* = 0.001, respectively).

The distribution of the EBNA1 subtypes among EBV Type1 and 2 in 108 lymphoma samples was presented in Table 3. There appears no significant association between the two classifications (*p* = 0.546).

**Table 3:** Association of EBNA1 subtypes and EBV types in the EBV isolates of lymphoma.

EBNA1 subtype	EBV type		
	Type 1	Type 2	Type 1+2
V-val	57(70.4%)	6(85.7%)	12(60%)
P-thrV	10(12.3%)	1(14.3%)	6(30%)
V-leuV	4 (4.9%)	0	0
P-ala	10(12.3%)	0	2(10%)
total	81(100%)	7(100%)	20(100%)

## Discussion

In the present study, we analyzed EBNA1 gene sequence in four kinds of EBV-associated lymphoma specimens (59 NK/T, 35 HL, 5 DLBCL, 11 T cell lymphoma) in Shandong province, Northern China. This has extended our previous report in gastric carcinomas and nasopharyngeal carcinomas [18] and expanded the spectrum of EBNA1 sequence variation in multiple tumors of this area. To our knowledge, this is the first report to have extensively explored the EBNA1 gene vatiations in lymphomas from a non-NPC endemic region in China.

Characterization of EBNA1 gene variations in various EBV-associated malignancies has been extensively studied, but a definitive conclusion about geographical and/or disease associations of EBNA1 subtype has not been reached. Controversial findings were reported by different groups. Some researchers proposed that a possible association exists between EBNA1 gene variation and tumors. Bhatia et al. [9] found V-leu subtype in 17/36 BL biopsies from Africa and North America and Gutiérrez et al.[10] detected this variant in 11/28 BL samples from Africa, North, and South America, but neither of them found V-leu variant in peripheral blood lymphocytes (PBLs) or oral secretions (OS) from healthy donors of that same regions. Therefore, they concluded that EBNA1 variation might influence the tissue tropism of EBV and contribute to varied tumorigenicity of the virus. However, others argued EBNA1 variations in favor of geographical restriction. Habeshaw et al.[14] analyzed EBNA1 variants in BL and control samples in three different geographic areas and found that V-leu was the most common EBNA1 sequence variant in endemic BL samples in East Africa (29 of 55 tumors) and it was also found in 18 of 32 control donors from the same area. In Europe, P-ala and P-thr were prevalent both in BL and control samples. So they concluded that in any geographic area, the EBNA1 subtypes reflected those EBV strains prevalent in the background population. Later reports reinforced the geographical association hypothesis. MacKenzie et al. [21] found V-leu preferentially in 12/20 lymphomas from Brazil and P-thr in 9/14 lymphomas from the United Kingdom. Chang et al. [22] reported 13/17 HL from Brazil present V-leu and 6/12 from the USA contained P-thr. Two other studies reported that 9/10 HL from Danish carried P-thr subtype and 9/19 lymphoma samples from Brazil showed V-leu variant respectively [15, 23]. Considering that P-thr or P-ala was prevalent strain in Europe and North America, while P-thr or V-leu was dominant in Africa and South America [17], these findings suggest that EBNA1 subtypes in tumor samples are similar to those in background population and may reflect geographical distribution of different virus strains.

In our present study, four EBNA1 subtypes: V-val, P-thrV, V-leuV and P-ala were observed. In each lymphoma group, the most common subtype was V-val without exception. Totally, V-val was found in 75 of 110 lymphoma samples (68.2%). These results were similar to our previous study on EBNA1 variants in NPC, EBVaGC and TW from healthy donors, in which V-val subtype was detected in 30 of 41 (73.2%) NPC, 32 of 41 EBVaGC (78.1%) and 34 of 55 (61.8%) TW [18]. In China, most studies focused on NPC and EBVaGC, in which V-val was the most common EBNA1 subtype [10, 12, 15, 16, 18, 24–26]. Lymhpoma is another malignancy tightly associated with EBV but there were only sporadic reports with a limited number of study subjects. Sandvej et al. [15] observed V-val subtype in 2/3 HL and 2/2 nasal NK/T cell lymphoma in Beijing, Northern China, and P-thr in the remaining 1 HD. Wang et al. [25] identified 2/2 HL, 1/2 T cell lymphoma, and 3/3 B cell lymphoma in Taiwan, Southern China, and a recent study showed that 33/34 children HL samples carried V-val subvariant [27]. Besides EBV-associated tumors, V-val was also found dominant in other EBV-positive samples of non-tumor EBV-associated diseases such as infectious mononucleosis (IM) [25, 27] and healthy donors [12, 15, 16, 18, 26]. These findings indicate that V-val is the prevalent EBV substrain in China.

In China, V-val subtype has been suggested to be oral tropic and preferentially associated with NPC [10, 12]. However, our previous study showed that V-val variant was not NPC-specific in Northern China[18] but only reflected geographical distribution of EBNA1 polymorphisms. Results from studies on EBVaGC gave the same conclusion as the latter [16]. We observed that the distribution of EBNA1 subtypes among the four different groups of lymphoma was not significantly different, and there was not a particular EBNA1 substrain associated with a certain subtype of lymphomas. Although the frequency of P-ala in lymphoma was a little higher than the other three EBV-positive samples, it couldn’t change the fact that V-val was also the predominant subtype in lymphoma. The significance of P-ala in lymphoma needs more extensive and profound exploration. Comparing EBNA1 variations in lymphoma with those in other EBV-positive samples, especially samples from healthy population in the same area, and the variation data in lymphoma samples from different geographical areas, we found that EBNA1 gene variations in lymphoma were similar to those in host background population and therefore support that conclusion that EBNA1 variations merely reflect the geographical distribution of different EBV strains but do not lead to any tumor specific association.

Traditionally, EBV has been divided into type 1 and type 2 substrains based upon sequence variations in EBNA2, EBNA 3A, 3B and 3C [20]. Type 1 strain is predominant throughout the world outside equatorial Africa and New Guinea [28] and has stronger transforming ability for resting B cells than type 2 strain [29]. In our present study, it was expected that type 1 was predominant accounting for 73.6% (81/110) of lymphoma samples. The distribution of EBV types in four kinds of lymphomas was similar, but different between lymphoma and NPC, EBVaGC or TW. The most notable difference was that there was higher frequency of coinfection with EBV type 1 and type 2 in lymphomas. Considering the fact that immunocompromised hosts are frequently infected with multiple viral strains [30], we speculated that the fact that lymphomas are malignant neoplasms derived from lymphatic system, which may greatly destroy the immune response, may be implicated in the different distribution of the mixed subtype between lymphoma and the other three groups. Strangely though, this was not the case for EBNA1 subtypes. There were only two lymphoma samples with dual EBNA1 substrains infection. This may attribute to the fact that EBNA1 subtypes are discordant with EBNA3C typing data, which was demonstrated in this study. Many investigations have been focused on relations of different EBV gene variations with EBV associated diseases [9, 21, 22, 31]. In the present study, we found no significant association between EBV subtypes and EBNA1 subtypes in lymphoma, which was different from our previous report in other tumor types [18]. In Habeshaw’s study [14], type 1 viruses were heterogeneous in terms of EBNA1 subtype, whereas type 2 viruses tended to be more uniform. This, along with our data, illustrates the complexity of the variant classifications and it is necessary to investigate relationships of various EBV gene variations with lymphomas to uncover the role of EBV in lymphoma pathogenesis.

Though we have not found evidence that EBV strains with certain EBNA1 variations contribute to pathogenesis of specific EBV-associated disorders, it is possible that the prevalence of particular EBNA1 subtype in various ethnic populations may play an important role in the varying incidence of various EBV-associated malignancies. V-val subtype is predominant in population from Asia, where NPC, EBVaGC and nasal NK/T cell lymphoma are more prevalent than in other geographical regions [32]. It has also been demonstrated that V-val subtype showed enhanced transcriptional activity and functional advantage when compared with the prototype B95-8 [26, 33]. This biological difference may result from the considerable sequence substitutions occurring in the functional domains of the carboxyl-terminus of EBNA1, including dimerization domain, DNA binding domain and transactivation domain [8, 34]. Though V-val subtype is most common both in Southern China and in Northern China, our findings are inconsistent with those reported from Southern China which demonstrated that V-val subtype of EBNA1 was preferentially associated with NPC. This contradiction reminds us not only more studies are needed but also the complex interplay between host genetic and environmental factors has to be taken into consideration besides the virus, when disentangling the pathogenesis of an EBV-associated disease.

In conclusion, rather than being tumor-specific, EBNA1 variants might only reflect the geographical restriction of EBV strains. Considering the discordance of various sub-typing methods, joint detection of sequence variations should be adopted. To further elucidate the role of EBNA1 variations in tumorigenesis, full scale epidemiological studies in different geographical areas should be performed to determine the distribution of EBNA1 subtypes in various EBV-associated tumors and control samples from the general population such as PBLs (peripheral B lymphocytes) and OS (oral secretion).
